# Supplementary material for: Hunger for Home Delivery: Cross-Sectional Analysis of the Nutritional Quality of Complete Menus on an Online Food Delivery Platform in Australia
Source: Nutrients. 2021 Mar 11;13(3):905. doi: 10.3390/nu13030905 (PMC8002002; doi:10.3390/nu13030905)
Supplement: Supplementary file 1 [file nutrients-13-00905-s001.pdf]

Table S1. Food Categories and their definitions with inclusions and exclusions.

Dairy and alternatives	Definition: Dairy and alternatives food group.<br>Includes: milk within dishes (e.g. milk on cereal), yoghurt, cheese and/or their alternatives
Breads and Cereals	Definition: Grain (cereal) food group, mostly wholegrain and or high cereal fibre varieties.<br>Includes: all breads and cereals (with or without fruit, nuts and seeds), pasta, noodles, roti, bread rolls, flat breads, oatmeal (porridge), crumpets<br>Excludes: Breakfast cereals categorised as discretionary (> 15g sugar per 100g)
Fruit	Definition: All fresh fruit, diced, canned fruit, stewed and dried.<br>Includes: oranges, mandarins and grapefruit, apricots, cherries, peaches, nectarines, plums, bananas, paw paw, mangoes, pineapple, melons, berries, grapes, passionfruit, apples, pears, watermelon, banana, coconut and guava<br>Excludes: fruit juice, which are coded under beverages
Vegetables	Definition: Vegetables food group – these can be fresh, frozen and canned vegetables.<br>Includes: green leafy or raw salad vegetables, sweet corn, potato or starchy vegetables, tomato, celery, sprouts, zucchini, squash, capsicum, eggplant, cucumber, okra, pumpkin, green peas, green beans, potato, cassava, sweet potato, taro, carrots, beetroot, onions, shallots, garlic, bamboo shoots, swede, turnip, broccoli, brussels sprouts, bok choy, cabbages, cauliflower, kale, lettuce, silverbeet, spinach and snow peas<br>Excludes: legumes
Vegetables (Other)	Definition: All other food items that are consumed like vegetables however botanically not a vegetable.<br>Includes: avocados, olives (of any variety), mushrooms, tomatoes, wakame (seaweed salad), chilli
Meat and Alternatives	Definition: Meat and alternatives food group.<br>Includes: Red meats such as beef, lamb, veal, pork, goat or kangaroo. Poultry such as chicken or turkey. Fish and seafood such as prawns, crab, lobster, mussels, oysters, scallops, clams. Eggs,tofu, Quorn etc.<br>Excludes: legumes, processed meats
Nuts and Seeds (incl. pastes)	Definition: Any nuts (or not botanically nuts e.g. Peanuts that are consumed like nuts) or seeds. These can be non-processed, roasted, peeled, salted or made into pastes.<br>Includes: Nuts such as almonds, pine nuts, walnut, macadamia, hazelnut, cashew, peanut, brazil nuts. Seeds such as pumpkin seeds, sesame seeds, sunflower seeds. Nut/Seed Pastes such as almond/peanut butter or tahini.<br>Excludes: Legume based dips
Legumes	Definition: Most legumes<br>Includes: All cooked, baked/roasted, dried or canned beans such as: red kidney beans, soybeans, lima beans, cannellini beans, chickpeas, lentils, split peas Legume based dips e.g. Hummus<br>Excludes: Soy based products e.g. Tofu that are classified under meat alternatives as sometimes it is difficult to distinguish between real and imitation meat<br>Peanuts as these are consumed more like nuts than legumes
Confectionery	Definition: Sweet discretionary products that are usually higher in sugar and/or fat.<br>Includes: Lollies, chocolate, roll-ups, nougat, fruit leather, sesame snaps, peanut brittle, chocolate coated fruit/nuts/seeds, chocolate hazelnut spreads, chocolate sauces
Bars	Definition: Product usually made from grains, whey protein, nuts and/or fruit. These can be packaged, homemade or made outside the home and purchased by the consumer.<br>Includes: Muesli bars, nut bars, protein bars/balls/biscuits/slices
Baked Goods/Desserts (homemade or similar)	Definition: These foods are usually made from flour. These may be made in an oven, microwave or on the benchtop and may have a confectionary style covering e.g. Frosting or chocolate. These are usually homemade or made outside the home by a bakery, café or dessert outlet and purchased by the consumer.<br>Includes: cookies, cakes, cake-type desserts, muffins, slices, sweet pies, scones, crumbles, iced buns, pancakes or crepes with/without toppings, waffles, soufflé, croissant, fruit crumbles, pastries<br>Excludes: bread<br>Examples: churros, baklava, gulab jamun, kanafeh, nutella pizzas
Discretionary snack food (Savoury) – Packaged	Definition: Packaged savoury snack foods that cannot be classified into other categories that are higher in one or more of the following: salt, sat fat, energy or added sugar.<br>Examples: potato chips, flavoured crisps and crackers, prawn crackers, popcorn, corn chips, pork crackling, pretzels, grain chips, cheese spread, rice crackers
Discretionary snack food (Sweet) - Packaged	Definition: Packaged sweet snack foods that cannot be classified into other categories that are higher in one or more of the following: salt, sat fat, energy or added sugar.

	Examples: biscuits with sweet fillings, wagon wheels, Timtams, Teevee snacks, yanyan, Pocky, Hello Panda etc.
Other snack food (other)	Other snack foods that do not belong to any other category<br>Includes: pickles, chewing gum
Iced confectionary and dairy-based desserts	Definition: Milk or water-based desserts that are cold and/or frozen.<br>Includes: Ice blocks, slushies, snow cones, jelly, frozen yoghurt, patbingsoo, and icecream, gelato, sundaes, sorbet, rice pudding, fromais frais, mousse, custard<br>che bau mau and similar Vietnamese iced drink desserts, Pannacotta
Processed meats	Definition: Meat that has been cured, salted, smoked or has had chemicals added to it.<br>Examples: sausage, bacon, ham, salami, luncheon meats
Cereal-based mixed meal (five food groups)	Definition: Mixed meals where cereal or cereal products are the major ingredients AND discretionary and/or processed meats are not an ingredient which is not clearly visible.<br>Examples: pasta, pizza, burgers, sandwiches, sushi, wraps, filled rolls, fried rice, fried noodles, ravioli, dumplings, pilaf, burrito, taco, congee (non-plain), steamed buns with fillings, nachos, risotto, gnocchi, fajitas<br>Pasta with tomato sauce, pizzas with core ingredients (no cheese) e.g. Meat Mannoush/ lahmacun, lahmajoun, lahme aiajine (multiple spellings). Noodle soups (pho, some chinese noodle soups). Burrito bowls with moderate amount of sauce.<br>Chicken and Vegetable Sandwiches/ wraps with sauce (unless excessive amounts of sauce).
Cereal-based mixed meal (Discretionary)	Definition: Meals where cereal or cereal products are the major ingredients, however are discretionary themselves i.e. Are higher in fat and saturated fat, sodium and sugar or contain visible discretionary products e.g. Bacon.<br>Examples: deep fried foods, take away pizza and burgers with processed meats, sushi with tempura (fried) filling, meat pies, quiche, sausage rolls, pizzas with >5g saturated fat/100g. Burgers with discretionary meats, brioche bun or mayo. Cream based/ rose sauce pasta, lasagne. Char Kway Teow, Japanese Ramen, other soy-sauce based noodle soups.
Cereal-based mixed meal (not further defined)	Definition: Meals where cereal or cereal products are the major ingredients and the proportion of five food group and discretionary cannot be easily discerned.
Meat or alternative based mixed meal (five food groups)	Definition: Meals where meat and alternatives are the major constituent AND discretionary and processed meats are not an ingredient which is clearly visible.<br>Examples: omelette, frittata, scrambled eggs, curry, stew, casserole, meatloaf, meat balls, rissoles, patties, crustless pies. Shish Kebab/ Souvlaki (using cubes of meat)<br>Meat + rice dishes (if not obvious what the serving is). Stirfry (if not obvious amount of sauce)
Meat or alternative based mixed meal (Discretionary)	Definition: Meat or meat-based products are the major ingredients, however are discretionary themselves i.e. Are higher in fat and saturated fat, sodium and sugar or contain visible discretionary products e.g. Bacon.<br>Examples: battered or crumbed fish and seafood, schnitzel, untrimmed meats (e.g. chicken wings)<br>Kebabs (Minced - kofta, kafta, seekh kebabs, adana (minced lamb) or shaved meat – Shawarma).<br>Coconut based sauce/soup/curry, satay sauce, paneer curries (containing creamy base e.g. using buttermilk or ghee), other cream-based curries, laksa, seafood salad with sauce<br>bifteki (Greek rissoles)
Meat or alternative based mixed meal (not further defined)	Definition: Meat or meat-based products are the major ingredients and the proportion of five food group and discretionary cannot be easily discerned.
Fats/Oils	Definition: Any fat-based spreads and/or oil.<br>Examples: Olive oil, canola, coconut oil, vegetable oil, sesame oil, butter or margarine
Fried Potato (or similar)	Definition: Any fried or oil cooked potato products including sweet potato.<br>Examples: hot chips (of any thickness, cut, size e.g. French fries, wedges, thick cut), hash browns, potato gems (or tatter tots).
Vegetable-based mixed meal (five food groups)	Definition: A meal in which vegetables are the major ingredients AND discretionary and processed meats are not an ingredient which is clearly visible.<br>Examples: salads with modest amount of dressings, vegetable-based curries, stews, or casseroles, vegetable patties, stir fries with or without meat/alternatives, dhal
Vegetable-based mixed meals (Discretionary)	Definition: A meal in which vegetables are the major ingredient, however are discretionary themselves i.e. Are higher in fat and saturated fat, sodium and sugar or contain visible discretionary products e.g. bacon<br>Examples: tempura vegetables, deep fried vegetable patties or croquettes, Caesar salad, falafel, creamy potato bakes, coleslaw
Vegetable-based mixed meal (not further defined)	Definition: A meal in which vegetables are the major ingredients and the proportion of five food group and discretionary cannot be easily discerned.

Soups	Definition: All broths, blended and chunky soups based from meat, fish, poultry or vegetables, containing a variety of ingredients.<br>Excludes noodle soups (e.g. Ramen, pho)
Special dietary foods	Definition: All other special dietary foods.<br>Includes: oral nutritional supplements (non-beverages only) e.g. puddings, jellies<br>Excludes: protein bars and supplemental vitamins (e.g. multivitamins, chewable Vitamin C and sish oil)
Prescription Medication and related	Definition: All prescription medication and tablets (e.g. multivitamins).
Sugar and Related Products	Definition: Any form of sweetener which can be added to foods.<br>Examples: honey, sugar (caster, cane, white, raw, powdered, etc.) syrups, nutritive and non-nutritive artificial sweeteners, jam.
Savoury Sauces, Condiments and Spreads	Definition: Any savoury sauce, condiment and non-fat spread.<br>Examples: tomato sauce, chutney, salad dressings, mayonnaise, vinegar, (non legume) dips and yeast spreads
Undetermined	Coder is not 100% certain of the food item/product.

Table S2. Beverage Categories and their definitions with inclusions and exclusions.

Beverage type	Example
Tea	Includes: All tea types (black, white, green, fruit tea) with minimal or no milk/sugar/honey at any temperature (hot/iced/warm).<br>Excludes: chain milk tea varieties e.g. pearl milk tea as these are high in sugar due to syrup used and will be considered a discretionary milk-based beverage or sugar sweetened beverages if no added milk
Coffee	Includes: All coffee types with minimal or no milk/sugar at any temperature (hot/iced/warm).<br>Excludes: coffee flavoured milk<br>Examples: macchiato, short black, Espresso, Greek Frappe
Water	Includes: All water types (sparkling, mineral, still, tap, filtered) and may contain added ingredients (e.g. lemon wedge, mint, dash of apple cider vinegar) that do not significantly alter the nutritional composition.<br>Excludes: tonic water<br>Example: Acqua panna (mineral water)
Juice	Includes: All fruit and vegetable juices, may be fresh, store purchased, cartooned with added or no added sugars. Includes coconut water (or juice)<br>Excludes: juices with any additional components e.g. milk
Energy Drinks	Includes: All beverages containing caffeine, guarana or any other similar stimulant compound.<br>Examples: Mother, V, Rockstar or Red Bull<br>Excludes: Coffee/Tea
Sugar Sweetened Beverages	Includes: All beverages containing added sugars and/or nutritive sweeteners.<br>Examples: soft drinks, cordial or non-dairy chain tea varieties.<br>Iced tea, Chinotto (Italian soft drink), Milkis (Korean Milk soft drink), Cascade (mixer), aloe vera (But if name has Juice, put in Juice), Cidona (Irish Softdrink), mineral water with added sugar, Lucozade (Japanese softdrink), Uludag (Turkish soft drink)
Non-Sugar Sweetened Beverages	Includes: All beverages without added sugars and/or nutritive sweeteners<br>Examples: diet soft drinks, zero sugar beverages, Nexba , kombucha
Water Based Flavoured Beverage – sugar not determined	Includes: Any beverage whose sugar level is not determined
Milk/Milk Alternatives	Includes: All animal milk of all fat levels, rice milk, almond milk, macadamia milk, soy milk, drinkable yoghurts, Ayran.<br>Excludes: any milk based beverages, yoghurt based bubble tea (e.g. yomie's yoghurt and rice drink)
Milk/Milk Alternative Based Beverages	Includes: Flavoured milk or milk/milk alternatives with additional core food items.<br>Examples: fruit smoothies, oak chocolate flavoured milk, iced coffee (without icecream or syrups), hot chocolate, milo, up and go, acai smoothie, piccolo latte, dirty chai, turmeric latte, cappuccino, latte, flat white<br>Excludes: plain milk/milk alternatives
Discretionary Milk Based Beverages	Includes: Milk/milk alternatives with additional discretionary items.<br>Examples: milk based drinks made with ice-cream, syrups and any other sweeteners e.g. chocolate milk shake with ice-cream/whipped cream or syrup, commercial milk teas with toppings (e.g. pearls)<br>frappe, chai latte, mocha, flavoured lattes (Taro, Matcha)<br>Excludes: plain milk/milk alternatives or milk/milk based beverages
Alcohol	Includes: All forms of alcohol.<br>Examples: Wine, whisky, beer, soju, cocktails etc.
Body Building and Performance Beverages	Includes: All sports based beverages – these are usually protein and/or amino acid based and consumed around (before/during/after) situations where the participant undertakes physical activity.<br>Examples: protein shakes (made in water or milk) or branched chain amino acids (BCAAs)<br>Excludes: all electrolyte drinks, these can be consumed for purposes other than physical activity
Rehydration Beverages (Electrolytes)	Includes: All electrolyte based drinks aimed to improve the hydration status of the consumer.<br>Examples: Gatorade, Powerade, Gastrolyte, HYDRAlyte, Pedialyte and Repalyte

Undetermined	Coder is not 100% certain of the food item/product.<br>Examples: bottles, can without description, kid's drink
Supplements	Includes: All other oral supplements.<br>Examples: fibre supplements or meal replacement beverages or carbohydrate gels for endurance runners<br>Excludes:sports beverages and rehydration beverages, meal replacement non-beverage items e.g. bars

Table S3. The proportion of food and beverage categories in the most popular menu items (n = 625) from 196 independent takeaways. Categories are sorted in descending order

Type of Category	Food Categories	n	%
Discretionary	Cereal-Based Mixed Meal	357	57.1
	Meat Or Alternative Based Mixed Meal	157	25.1
	Vegetable-Based Mixed Meal	20	3.2
	Fried Potato (Or Similar)	16	2.6
	Baked Goods/Desserts (Homemade Or Similar)	8	1.3
	Iced Confectionary And Dairy-Based Desserts	3	0.5
	Other Food[a]	3	0.5
	Discretionary Milk Based Beverages	2	0.3
	Savoury Sauces, Condiments And Spreads	2	0.3
	Other Beverage[b]	0	0
	Sugar Sweetened Beverages	0	0
Five Food Groups	Cereal-Based Mixed Meal	22	3.5
	Meat Or Alternative Based Mixed Meal	19	3
	Vegetable-Based Mixed Meal	15	2.4
	Other Food[e]	1	0.2
	Juice	0	0
	Other Beverage[d]	0	0
	Water	0	0
	**Total**	625	
